# The Role of the VWF/ADAMTS13 Axis in the Thromboinflammatory Response in Ischemic Stroke After SARS‐CoV2 Infection

**DOI:** 10.1002/brb3.70348

**Published:** 2025-02-19

**Authors:** Nadim Luka, Kieron South, Rachel Jones, Amanda J. Unsworth, Graham Coutts, Ioana‐Emilia Mosneag, Mehwish Younas, Amy Bradley, Siew Yan Wong, Ellen Collins, Chloe Quigley, Sean B. Knight, Barry W. McColl, Laura McCulloch, John R. Grainger, Craig J. Smith, Stuart M. Allan

**Affiliations:** ^1^ Geoffrey Jefferson Brain Research Centre, School of Biological Sciences, Faculty of Biology, Medicine and Health The University of Manchester Manchester UK; ^2^ Division of Neuroscience The University of Manchester Manchester UK; ^3^ Division of Cardiovascular Sciences The University of Manchester Manchester UK; ^4^ Lydia Becker Institute of Immunology and Inflammation, Faculty of Biology, Medicine and Health Manchester Academic Health Science Centre, The University of Manchester Manchester UK; ^5^ Discovery and Translational Science Department, Leeds Institute of Cardiovascular and Metabolic Medicine University of Leeds Leeds UK; ^6^ Geoffrey Jefferson Brain Research Centre, Manchester Academic Health Science Centre, Northern Care Alliance NHS Foundation Trust University of Manchester Manchester UK; ^7^ Division of Immunology, Immunity to Infection and Respiratory Medicine The University of Manchester Manchester UK; ^8^ UK Dementia Research Institute, Centre for Discovery Brain Sciences The University of Edinburgh Edinburgh UK; ^9^ Centre for Inflammation Research, Institute for Regeneration and Repair The University of Edinburgh Edinburgh UK

**Keywords:** ADAMTS13, ischemic stroke and thromboinflammation, SARS‐CoV2, VWF

## Abstract

**Background:**

SARS‐CoV2 infections increase the risk of ischemic stroke (IS), potentially through a thromboinflammatory cascade driven by an imbalance in the ratio of Von Willebrand Factor (VWF) and a disintegrin and metalloproteinase with a thrombospondin type 1 motif, member 13 (ADAMTS13), leading to the formation of ultra‐large VWF (UL‐VWF). However, the SARS‐CoV2 infection's contribution to any VWF/ADAMTS13 axis imbalance and the subsequent thromboinflammatory response post‐stroke remain poorly understood.

**Methods:**

We performed a detailed thromboinflammatory profile of the plasma samples from three experimental cohorts matched by age, sex, and stroke severity: non‐stroke controls (*n* = 23), SARS‐CoV2 negative IS (*n* = 22), and SARS‐CoV2 positive IS (*n* = 24). SARS‐CoV2 positive IS patients presented varying degrees of infection severity.

**Results:**

We observed an increase in VWF and UL‐VWF and a decrease in ADAMTS13 in the SARS‐CoV2 positive IS cohort, suggesting a VWF/ADAMTS13 axis imbalance. Interleukin‐6 (IL‐6) levels were positively correlated with VWF and negatively correlated with ADAMTS13, suggesting that IL‐6 may drive this imbalance. Fibrinogen and D‐Dimers were elevated in SARS‐CoV2 negative IS cohort and SARS‐CoV2 positive IS cohort, but D‐Dimers were within the normal range, indicating no disseminated intravascular coagulation. Factor IX (FIX) was elevated in the SARS‐CoV2 negative IS cohort. Tissue plasminogen activator (tPA) was elevated in the SARS‐CoV2 positive IS cohort, suggesting no fibrinolysis defects. Matrix Metalloproteinase‐2 (MMP‐2) and soluble Intracellular Adhesion Molecule‐1 (sICAM‐1) were elevated in the SARS‐CoV2 negative IS cohort.

**Conclusions:**

We show that SARS‐CoV2 infections drive a VWF/ADAMTS13 axis imbalance, inducing an increase in tPA while decreasing FIX, MMP‐2, and sICAM‐1 post‐stroke.

## Graphical abstract

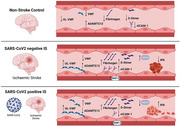

## 1 Introduction

Ischemic stroke (IS) is a predominant cause of mortality and morbidity worldwide (King et al. 2020). Historically, respiratory tract infections (RTIs) have been associated with a greater likelihood of developing coagulopathies and an increased risk of IS (Paganini‐Hill et al. 2003). Additionally, there is an increasing body of evidence indicating that an RTI preceding IS worsens the outcome (Oh and Parikh 2022; Takacs et al. 2023), which has been further highlighted by the COVID‐19 pandemic (Knight et al. 2022; Perry et al. 2021). However, the molecular mechanisms involved in the worsening of the outcome of IS after an RTI are yet to be fully elucidated.

Thromboinflammation is a vascular process that regulates the interactions between coagulation and inflammation (Vagionas et al. 2022). The thromboinflammatory response is strongly driven by the release of Von Willebrand Factor (VWF), a multimeric blood glycoprotein involved in hemostasis. VWF is solely expressed in endothelial cells and megakaryocytes and is stored in the Weibel–Palade bodies (WPB) of the former (Schick et al. 1997). In cases of vascular damage triggered by endothelial dysfunction or inflammation, activation of endothelial cells exposes VWF, which accumulates in the subendothelial matrix, and promotes the formation of ultra‐large VWF (UL‐VWF) (Denorme et al. 2019). Under shear stress conditions VWF promotes platelet adhesion and activation (Arce et al. 2021) and it has also been reported to play a key role in endothelial leukocyte recruitment upon inflammation (Petri et al. 2010). UL‐VWF is proteolytically cleaved, into smaller and less reactive multimers of VWF, by the plasma protease ADAMTS13 (a disintegrin and metalloproteinase with thrombospondin type 1 motif, 13) (Masias and Cataland 2018).

The homeostatic relationship between VWF and ADAMTS13 is referred to as the VWF/ADAMTS13 axis. Imbalance of the VWF/ADAMTS13 axis results from increased levels of VWF and decreased levels of ADAMTS13 (Thangaraju et al. 2021), which exacerbates the thromboinflammatory response and in turn causes hypercoagulation (Peyvandi et al. 2011), inhibition of fibrinolysis (Abdul et al. 2017), neutrophil activation, and interaction with neutrophil extracellular traps (NETs) DNA (Grassle et al. 2014). Clinical data on patients with SARS‐CoV2 infection show VWF/ADAMTS13 axis imbalance that is proportional to the COVID‐19 severity. Furthermore, this imbalance was associated with an increased level of thrombin in SARS‐CoV2 patients, indicating a systemic hypercoagulative state (Thangaraju et al. 2021; Mancini et al. 2021). Similarly, IS has been shown to promote the imbalance of the VWF/ADAMTS13 axis in clinical samples (Taylor et al. 2020), and VWF‐deficient mice undergoing experimental IS show decreased infarct size and reduced neutrophil recruitment, to the infarct area (Denorme et al. 2021). Furthermore, mice treated with a pharmacologically constitutively active ADAMTS13 after experimental stroke also show smaller infarct size, reduced neutrophil recruitment and reduced microglial activation (South et al. 2022). The thromboinflammatory response mediated by the VWF/ADAMTS13 axis imbalance therefore plays a pivotal role in the progression of both SARS‐CoV2 infection and IS pathophysiology. However, the role of SARS‐CoV2 in the thromboinflammatory response post‐stroke has not been studied. We therefore aimed to investigate how preceding SARS‐CoV2 infection altered changes in thromboinflammatory factors in patients with IS.

## 2 Material and Methods

### 2.1 Study Design

#### 2.1.1 SARS‐CoV2 Positive IS Cohort

Patients were prospectively recruited between May 19, 2020 and March 10, 2021 at Manchester Centre for Clinical Neurosciences (MCCN), Salford Royal Foundation NHS Trust (SRFT). Informed consent was obtained from all patients, and clinical data were collected from medical records. Patients with confirmed IS who tested positive for SARS‐CoV2 by reverse transcriptase polymerase chain reaction (RT‐PCR) of nasopharyngeal swabs were enrolled in the study. The severity of the stroke was assessed using the National Institutes of Health Stroke Scale (NIHSS) score. The severity of the SARS‐CoV2 infection was defined by the degree of respiratory failure and/or radiological findings as previously described (Mann et al. 2020). Very mild cases were characterized by the absence of oxygen supplementation and no indication of COVID‐19‐related pneumonitis in the radiological data. Mild cases were characterized by supplemental oxygen requirement of either <3 L or <28%; alternatively, mild patients were characterized by not requiring any oxygen supplementation but showing indications of COVID‐19‐related pneumonitis radiologically. Moderate cases were characterized by supplemental oxygen requirement of <10 L or <60%. Severe cases were characterized by a supplemental oxygen requirement of >10 L or >60%.

#### 2.1.2 SARS‐CoV‐2 Negative IS Cohort

Patients with IS without evidence of SARS‐CoV2 were prospectively recruited from August 6, 2021 to September 23, 2022 at MCCN. Informed consent was obtained from all patients, and clinical data were collected from medical records. Patients were tested for SARS‐CoV2 infection by RT‐PCR to ensure that they were negative upon admission. NIHSS was used to assess the severity of the stroke.

#### 2.1.3 Non‐Stroke Controls

Non‐stroke controls were collected as part of a previous study pre‐dating the COVID‐19 pandemic. The non‐stroke controls were sex‐ and age‐matched to the SARS‐Cov2 positive IS cohort and to the SARS‐CoV2 negative IS cohort.

#### 2.1.4 Plasma Preparation

Whole blood was collected at a median time of 2 days for the SARS‐CoV2 negative IS cohort and 3.5 for the SARS‐CoV2 positive IS cohort after onset of the IS. The whole blood was collected using a S‐monocuvette tube (Sarstedt) containing 1.6 mg/mL K3 ethylenediaminetetraacetic acid (EDTA). The samples were centrifuged at 2000 *g* for 10 min at 4°C. The plasma was collected and stored at −80°C.

### 2.2 LEGENDplex Assay

Inflammation, fibrinolysis, vascular inflammation, and thrombosis markers were quantified using LEGENDplex custom panels (Biolegend). A human thrombosis 7‐plex (tissue plasminogen activator (tPA), D‐Dimer, plasminogen activator inhibitor (PAI)‐1, Factor IX (FIX), soluble cluster of differentiation (sCD) 40L, P‐selectin, and P‐selectin glycoprotein ligand‐1 (PSGL‐1)), a custom human thrombosis 3‐plex (tissue factor (TF)), a human fibrinolysis 5‐plex panel (plasminogen, antithrombin, prothrombin, Factor XIII (FXIII), and fibrinogen), and a custom human vascular inflammation 12‐plex panel (matrix metalloproteinase (MMP)‐2, MMP‐9, soluble vascular cell‐adhesion molecule (sVCAM)‐1, soluble intracellular cell‐adhesion molecule (sICAM)‐1, and myeloperoxidase (MPO)) were used for the analysis. The different panels were performed according to the manufacturer's instructions. The assays were analyzed using a BD Symphony flow cytometer (BD Biosciences). The collected data were analyzed using Qognit LEGENDplex (Biolegend) data analysis software.

### 2.3 Quantification of VWF/ADAMTS13 Axis Markers in Plasma by ELISA

VWF and ADAMTS13 antigen levels were measured using previously described in‐house ELISA (Andersson et al. 2012, Chion et al. 2007). UL‐VWF was quantified using a collagen binding assay (CBA) (Newnham et al. 2019). The results are shown as the ratio of VWF: CBA/Ag, which represents the ratio of the VWF bound to collagen (VWF: CBA (%)) over the total VWF antigen concentration (VWF: Ag) normalized to non‐stroke controls.

### 2.4 IL‐6 ELISA

The plasma concentration of IL‐6 was quantified using an ELISA (R&D Systems). The assay was performed according to the manufacturer's instructions.

### 2.5 Statistical Analysis

All data were analyzed with GraphPad Prism 9.1.2 (GraphPad Software Inc). The equal variation and normality of the data was assessed using the Shapiro–Wilk test. A one‐way analysis of variance (ANOVA) was followed with a Kruskal–Wallis test in non‐normal distributions. The data are presented as mean ± standard deviation (SD). The statistical significance is presented as *p < 0.05, **p < 0.01, ***p < 0.001, and ****p < 0.0001.

## 3 Results

### 3.1 Participant Characteristics

A total of 69 participants were recruited as follows: non‐stroke control cohort (*n* = 23), SARS‐CoV2 negative IS cohort (*n* = 22), and SARS‐CoV2 positive IS cohort (*n* = 24). In the SARS‐CoV2 positive IS cohort, the majority of the SARS‐CoV2 were either very mild (*n* = 8) or mild (*n* = 8); the remaining patients had either moderate (*n* = 5) or severe (*n* = 3) infections. The three cohorts were matched as closely as possible for age, biological sex, and stroke severity in the case of the stroke cohorts. The non‐stroke control cohort had a median age of 69 years, and 52.2% were males. The SARS‐CoV2 negative IS cohort had a median age of 71 years, and 63.6% of patients were males. The SARS‐CoV2 positive cohort had a median age of 73 years, and 62.5% of patients were males (Table 1). The SARS‐CoV‐2 negative IS cohort had a median NIHSS of 5.5, and the SARS‐CoV2 positive IS cohort had a median NIHSS of 5 (Table 1). The plasma from patients was collected with a median time of 2 days for the SARS‐CoV2 negative IS cohort and 3.5 days for the SARS‐CoV2 positive IS cohort. The SARS‐CoV2 infection was confirmed by RT‐PCR in the SARS‐CoV2 positive IS cohort.

**TABLE 1:** Clinical characteristics of the experimental cohorts. The table outlines the demographics, stroke treatment, cardiovascular co‐morbidities, and medications information of the different patients enrolled in the study. The data are median (IQR) and *n* (%), where *N* is the number of patients present in each cohort. N/A: Not applicable.

	Non‐stroke controls	SARS‐CoV2 negative IS	SARS‐CoV2 positive IS
***n***	23	22	24
**Age (years)**	69 (53–74)	71 (61–74)	73 (65–80)
**Sex (male)**	12/23 (52.2%)	14/22 (63.6%)	15/24 (62.5%)
**Illness onset to sample (days)**	N/A	2 (1.2–2.5)	3.5 (3–4)
**Clinical information**			
**NIHSS**	N/A	5 (4–9.5)	5 (4–11)
**SARS‐CoV2 infection severity**			
**Very mild**	N/A	N/A	8/24 (33.3%)
**Mild**	N/A	N/A	8/24(33.3%)
**Moderate**	N/A	N/A	5/24 (20.8%)
**Severe**	N/A	N/A	3/24 (12.5%)
**Stroke treatment**			
**Thrombolysis**	N/A	7/22 (31.8%)	2/24 (8.3%)
**Thrombectomy**	N/A	3/22 (13.6%)	1/24 (4.2%)
**Co‐morbidity**			
**Hypertension**	6/23 (26%)	8/22 (36.4%)	13/24 (54.2%)
**Coronary heart disease**	5/23 (21.7%)	3/22 (13.6%)	7/24 (29.2%)
**Diabetes mellitus**	3/23 (13.1%)	6/22 (27.3%)	7/24 (29.2%)
**Dyslipidaemia**	11/23 (47.8%)	13/22 (59.1%)	17/24 (70.3%)
**Atrial fibrillation**	1/23 (4.3%)	13/22 (13%)	7/24 (29.2%)
**Smoking**	11/23 (47.2%)	11/22 (50.0%)	10/24 (41.7%)
**Medications**			
**Antiplatelets**	5/23 (21.7%)	6/22 (27.3%)	10/24 (41.7%)
**Anticoagulants**	0/23 (0.0%)	0/22 (0.0%)	6/24 (25.0%)
**ACE Inhibitors**	3/23 (13.0%)	4/22 (18.2%)	6/24 (25.0%)
**Statin**	9/23 (39.1%)	13/22 (59.1%)	18/24 (75.0%)
**Angiotensin receptor blocker**	2/23 (8.7%)	1/22 (4.6%)	2/24 (8.3%)
**β‐Blockers**	5/23 (21.7%)	5/22 (22.7%)	10/24 (41.7%)

### 3.2 VWF/ADAMTS13 Axis Imbalance in the SARS‐CoV2 Positive IS Cohort

**FIGURE 1:**
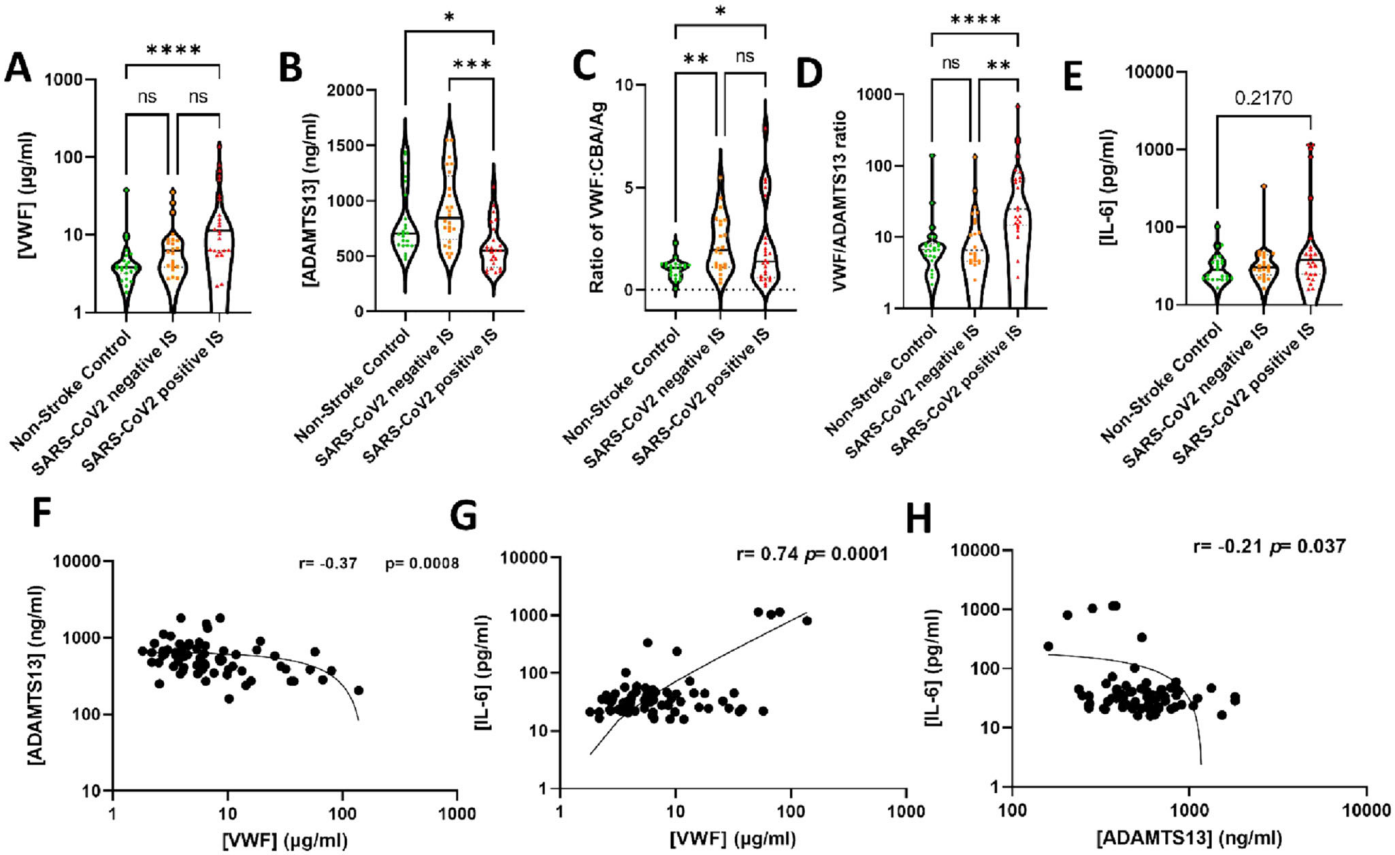
Plasma from SARS‐Cov2 positive IS patients show an imbalance in the VWF/ADAMTS13 axis. The plasma concentration of (A) VWF and (B) ADAMTS13 was quantified by in‐house ELISA. (C) UL‐VWF levels were determined by collagen binding assay UL‐VWF was normalized to the Non‐stroke control cohort and to the total VWF antigen levels to obtain a ratio of CBA/Ag. (D) The VWF/ADAMTS13 imbalance was also measured by the ratio of VWF levels over ADAMTS13 levels. (E) Plasma levels of IL‐6 were measured using a commercial ELISA. A Spearman correlation analysis was performed to investigate the relationship between VWF and ADAMTS13 (F), the relationship between IL‐6 with (G) VWF, and (H) ADAMTS13. Each data point represents the mean of the duplicate concentration calculated. The results are shown as the mean ± standard deviation (SD). The statistical analysis between the experimental groups was performed using Kruskal–Wallis test.*p < 0.5, ***p < 0.001, and ****p < 0.0001.

The SARS‐CoV2 positive IS cohort showed elevated plasma levels of VWF compared to both the non‐stroke control cohort and the SARS‐CoV2 negative IS cohort (Figure 1A). Plasma levels of ADAMTS13 were within the normal range for the non‐stroke control cohort and SARS‐CoV2 negative IS cohort, while they were significantly decreased in the SARS‐CoV2 positive IS cohort. UL‐VWF levels were determined using a CBA (Figure 1B). This assay measured the ratio of collagen‐bound VWF (VWF: CBA) over the total antigen levels of VWF (VWF: Ag) normalized to the non‐stroke controls. The SARS‐CoV2 negative IS and SARS‐CoV2 positive IS cohorts showed increased levels of UL‐VWF compared to the non‐stroke control cohort (Figure 1C). Furthermore, the SARS‐CoV2 positive IS showed an elevated VWF/ADAMTS13 ratio (Figure 1D). Overall, a VWF/ADAMTS13 axis imbalance was observed in the SARS‐CoV2 positive IS cohort.

In addition, plasma levels of inflammatory cytokines were measured. IL‐6 was elevated in the SARS‐CoV2 positive IS cohort, but the increase was not statistically significant (Figure 1E). The levels of the other inflammatory cytokines were unchanged among the different experimental cohorts. A significant but modest negative correlation was observed between the plasma concentration of VWF and that of ADAMTS13 (Figure 1F). Furthermore, the plasma levels of VWF and IL‐6 were strongly and significantly positively correlated (Figure 1G). Conversely, the plasma concentration of ADAMTS13 and IL‐6 exhibited a significant negative correlation (Figure 1H).

### 3.3 Hypercoagulative State in IS and SARS‐CoV2 Positive IS

**FIGURE 2:**
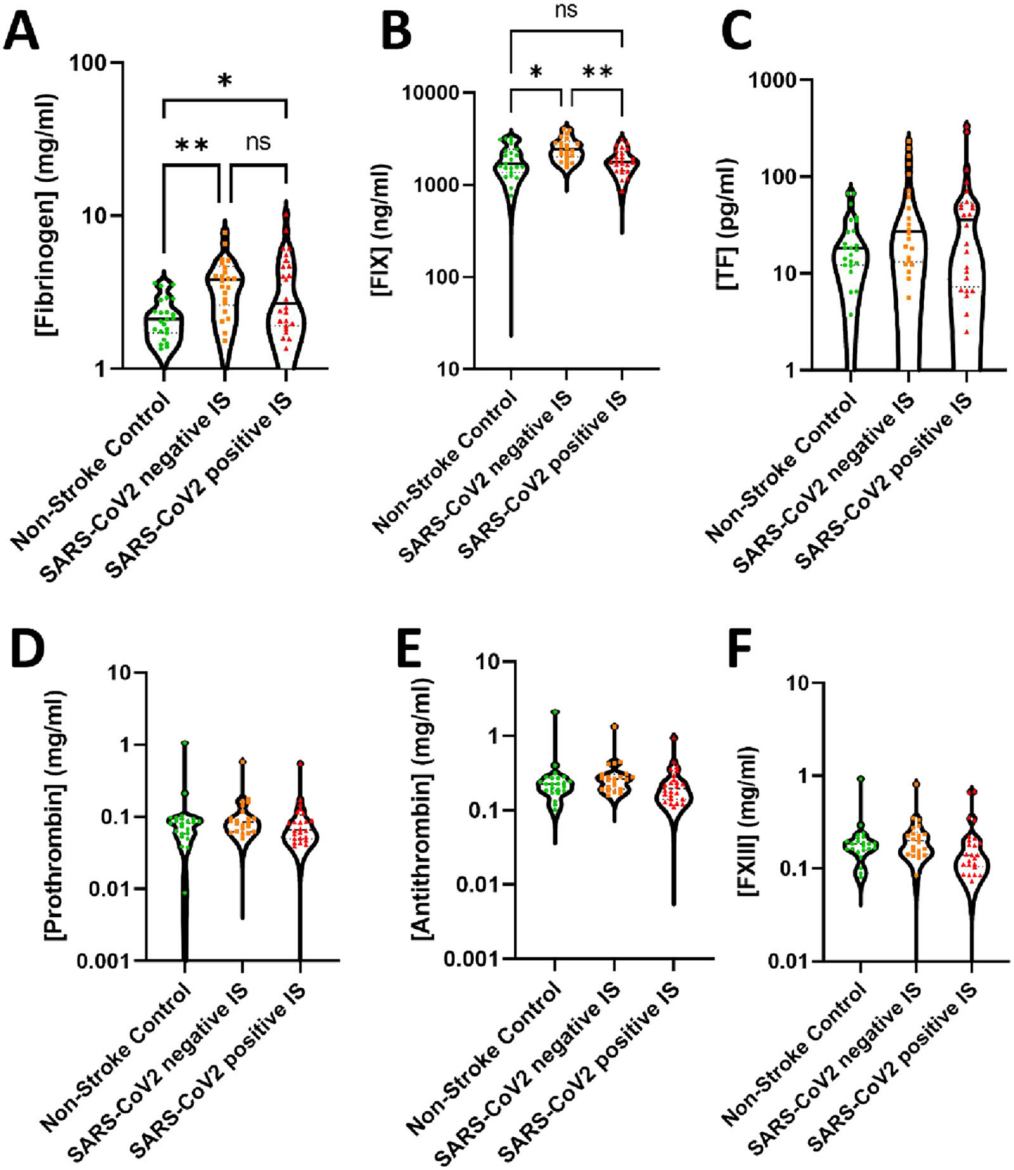
A hypercoagulative state in both ischemic stroke cohorts. The plasma concentration of (A) fibrinogen, (B) Factor IX (FIX), (C) tissue factor (TF), (D) prothrombin, (E) antithrombin, and (F) Factor XIII (FXIII) was measured using LEGENDplex assay (human fibrinolysis 5‐plex and human thrombosis 7‐plex panels). Each data point represents the mean of the duplicate concentration calculated. The results are shown as the mean ± standard deviation (SD). The statistical analysis between the experimental cohorts was performed using a Kruskal–Wallis test. *p < 0.5 and **p < 0.01.

Plasma levels of fibrinogen were elevated in the SARS‐CoV2 negative IS cohort and SARS‐CoV2 positive IS cohort compared to the non‐stroke control cohort. However, the infection preceding the stroke did not affect the plasma levels of fibrinogen (Figure 2A). Interestingly, the plasma levels of FIX were elevated in the SARS‐CoV2 negative IS cohort but remained unchanged in the SARS‐CoV2 positive IS cohort (Figure 2B). Tissue factor levels were elevated in the SARS‐CoV2 negative IS cohort and the SARS‐CoV2 positive IS cohorts, but this increase was not statistically significant (Figure 2C). Prothrombin, antithrombin, and FXIII remained unchanged in all cohorts (Figure 2D–F).

### 3.4 Elevated Fibrinolysis Markers in SARS‐CoV2 Positive IS

**FIGURE 3:**
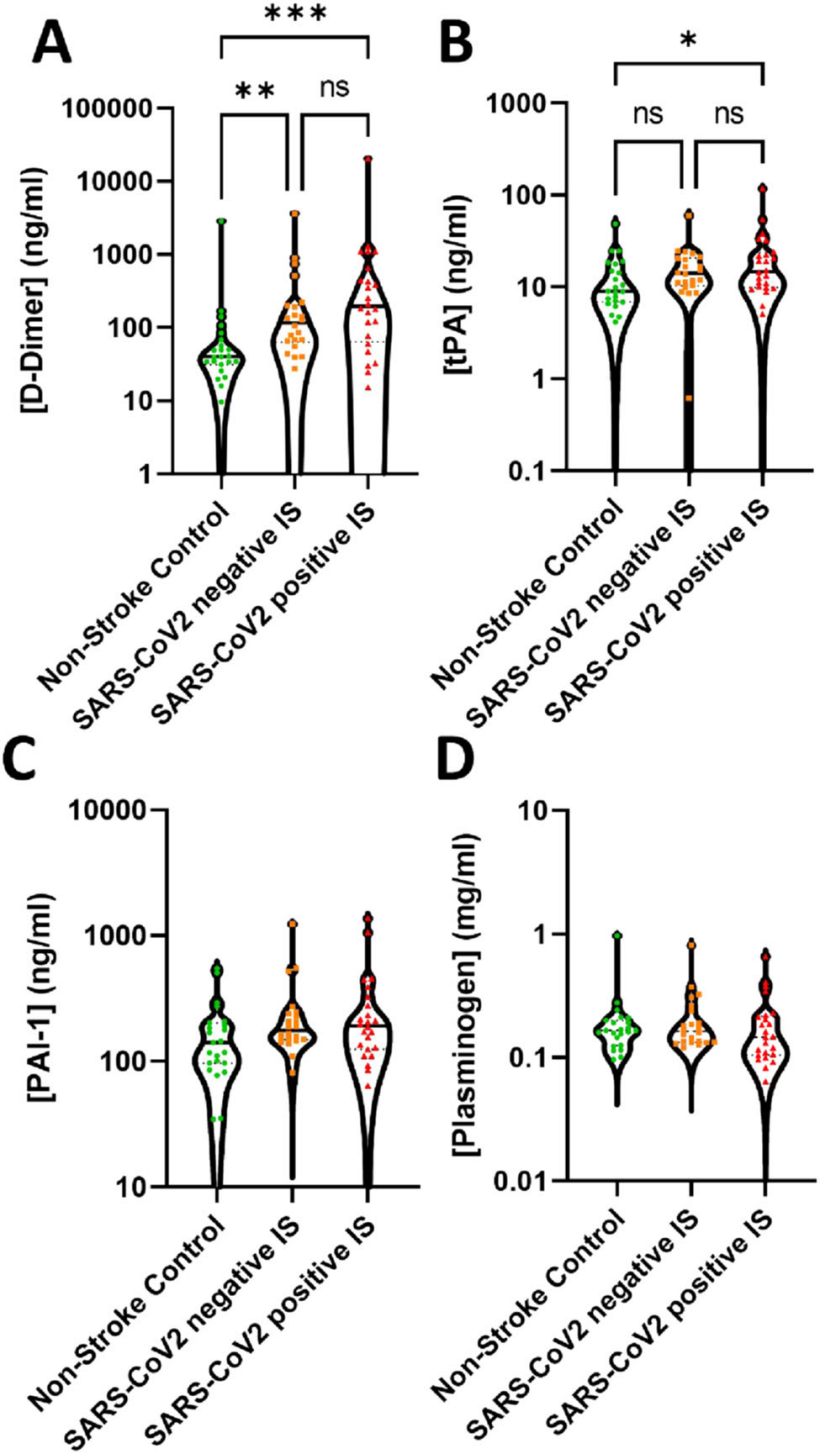
Fibrinolysis was not inhibited by the IS or the infection. The plasma concentration of (A) D‐Dimers, (B) tissue plasminogen activator (tPA), (C) Plasminogen activator inhibitor‐1 (PAI‐1), and (D) plasminogen was measured using LEGENDplex assay (human fibrinolysis 5‐plex and human thrombosis 7‐plex panels). The results are shown as the mean ± standard deviation (SD). The statistical analysis between the experimental cohorts was performed using a Kruskal–Wallis test. *p < 0.5, **p < 0.01, and ***p < 0.001.

D‐Dimer levels were found to be elevated in the SARS‐CoV2 negative IS cohort and SARS‐CoV2 positive IS cohorts (Figure 3A). tPA was elevated in the SARS‐CoV2 positive IS cohort (Figure 3B). Plasma levels of PAI‐1 and plasminogen were unchanged (Figure 3C,D).

### 3.5 Increased Levels of MMP‐2 and sICAM‐1 in the SARS‐CoV2 Negative IS Cohort

**FIGURE 4:**
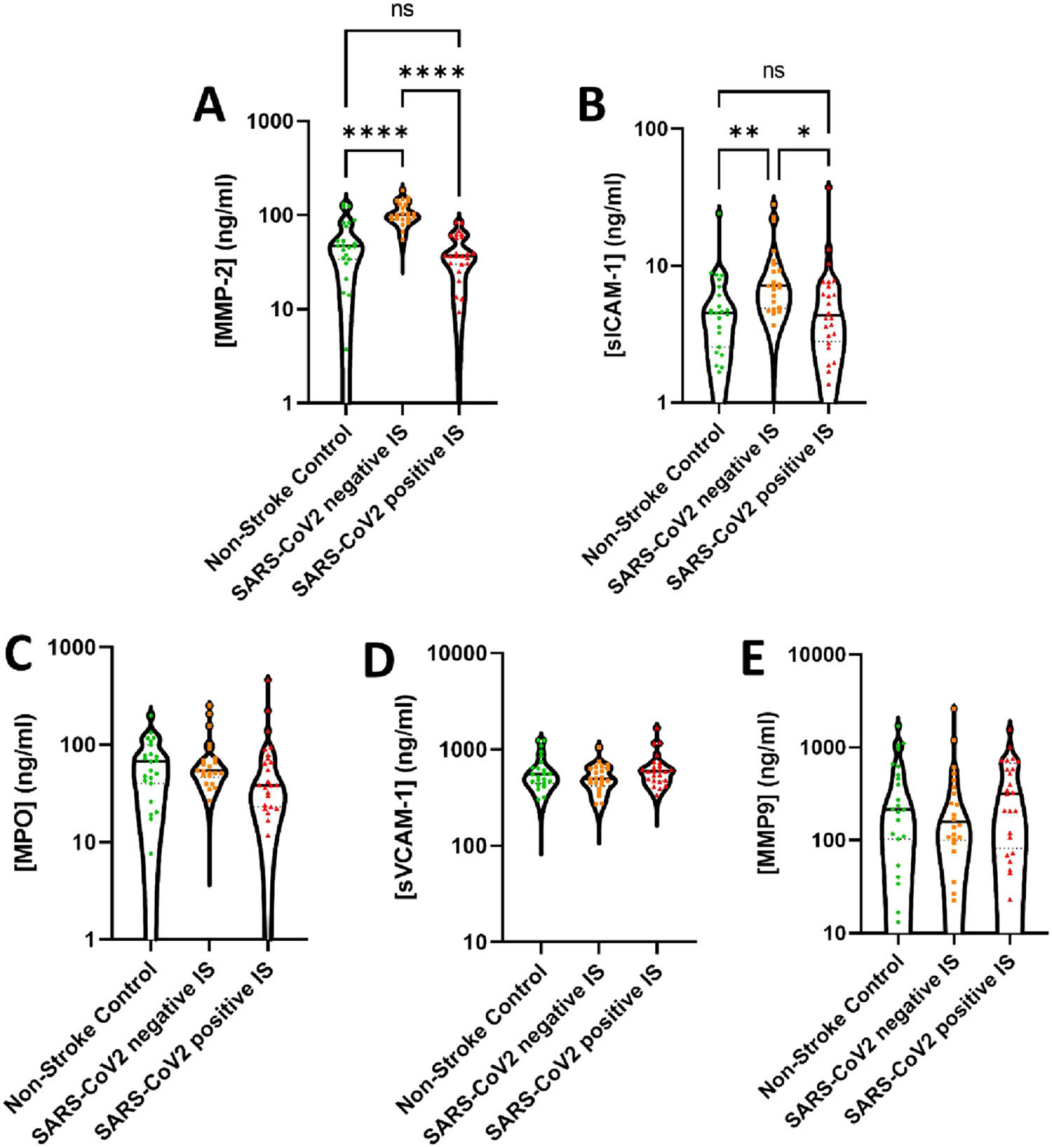
Vascular inflammation driven by MMP2 and ICAM‐1 in the SARS‐CoV2 negative IS cohort. The plasma concentration of (A) matrix metallopeptidase‐2 (MMP‐2), (B) soluble intercellular adhesion molecule‐1 (sICAM‐1), (C) myeloperoxidase (MPO), (D) soluble vascular cell adhesion molecule‐1 (sVCAM‐1), and (E) MMP‐9 was measured using LEGENDplex assay (human custom vascular inflammation 12‐plex panel). The results are shown as the mean ± standard deviation (SD). The statistical analysis between the experimental cohorts was performed using a Kruskal–Wallis test. *p < 0.5, **p < 0.01 and ****p < 0.0001.

Finally, the plasma concentrations of MMP‐2 and sICAM‐1 were elevated in the SARS‐CoV2 negative IS cohort. For the SARS‐CoV2 positive IS cohort, MMP‐2 and sICAM‐1 returned to levels similar to the non‐stroke control cohort (Figure 4A,B). Plasma levels of MPO, MMP‐9, and sVCAM‐1 remained unchanged in all experimental cohorts (Figure 4C–E).

## 4 Discussion

Here, we show that SARS‐CoV2 infection preceding IS induces thromboinflammatory changes compared to IS without preceding infection and non‐stroke controls, respectively. Most notably, SARS‐CoV2 infection exacerbates the imbalance in the VWF/ADAMTS13 axis seen post‐IS. Additionally, the infection prior to IS does not elevate plasma protein levels of MMP‐2, sICAM‐1, and FIX to the same extent as IS without a preceding infection. Finally, the SARS‐CoV2 infection preceding IS does not appear to affect fibrinolysis post‐IS.

In the current study, we have observed increased plasma levels of VWF in the SARS‐CoV2 positive IS cohort. VWF in human plasma has been identified as a key driver of a hypercoagulative state that is associated with the outcome and severity in both SARS‐CoV2 (Ladikou et al. 2020) and IS (Taylor et al. 2020) patients. These observations agree with our findings in the SARS‐CoV2 positive IS cohort, but not with the SARS‐CoV2 negative IS data. This may indicate an additive effect between the SARS‐CoV2 infection and the IS. We also report a positive correlation between plasma levels of IL‐6 and VWF. The release of VWF is dependent on its release from the WPB, which occurs upon endothelial activation and is promoted by IL‐6 (Chi et al. 2001).

We have also observed that the antigen levels of ADAMTS13 decreased in the SARS‐CoV2 positive IS cohort but remained unchanged in the SARS‐CoV2 negative IS cohort. Previous studies report that ADAMTS13 antigen levels and activity substantially reduced in SARS‐CoV2 infections (Mancini et al. 2021; Hafez et al. 2022) and in patients following a severe IS (Denorme et al. 2017). The latter is conflicting with our data and may be explained by the mild nature of the IS in our cohorts. However, to our knowledge, this represents the first report indicating that a mild SARS‐CoV2 infection and, more generally, an RTI preceding a mild IS causes a robust thromboinflammatory imbalance, thus inducing a systemic decrease in ADAMTS13 plasma levels. We have also shown that IL‐6 is negatively correlated to ADAMTS13, which may indicate a suppressive role of ADAMTS13 by IL‐6. An inhibitory role of IL‐6 on ADAMTS13 has been reported in vitro (Bernardo et al. 2004). The negative correlation between ADAMTS13 and VWF is consistent with the known physiological role of the VWF/ADAMTS13 axis (Muia et al. 2014), but the relationship between IL‐6 with ADAMTS13 and VWF suggests that IL‐6 could regulate the balance of the VWF/ADAMTS13 axis.

We also conducted a comprehensive screening of the coagulation factors involved in the coagulation cascade. Fibrinogen was found to be elevated in the SARS‐CoV2 negative IS cohort and SARS‐CoV2 positive IS cohort with slightly higher than normal levels of >3.5 mg/mL (Di Napoli and Singh 2009). Studies have shown that elevated fibrinogen levels are associated with cognitive decline (Liu et al. 2019) and negative functional outcomes (Hou et al. 2021) post‐IS. Furthermore, increased levels of fibrinogen have been observed in patients affected by SARS‐CoV2 infection and were associated with the infection severity (Sui et al. 2021). Fibrinogen has been additionally linked with post‐stroke‐associated *Streptococcus pneumoniae* infections (Lin et al. 2021). Fibrinogen is a coagulation factor involved in the formation of fibrin, an essential precursor for a stable fibrin clot, platelet activation, and aggregation. In addition, fibrinogen is also an important inflammation marker. The elevated fibrinogen levels indicate that the SARS‐CoV2 positive IS and SARS‐CoV2 negative IS cohorts are more prone to a hypercoagulative and inflammatory environment post‐stroke. Interestingly, FXIII, which is important in fibrin stabilization and cross‐linking, remained unchanged in both IS cohorts. These results are in contrast with data indicating that high FXIII levels are a predictor for unfavorable outcomes of SARS‐CoV2 infection (Marchetti et al. 2022) and IS (Zhang et al. 2021). The hypercoagulative state was most predominant in the SARS‐CoV2 negative IS cohort, which showed elevated levels of both FIX and D‐dimer that may indicate disseminated intravascular coagulation (DIC) (Yang et al. 2014). However, in our study D‐Dimer content was within the clinically normal concentration (<500 ng/mL). Hence, this suggests that there is no significant DIC in SARS‐CoV2 negative IS and SARS‐CoV2 positive IS patients. SARS‐CoV2 has been reported to show increased DIC in severe patients but not in milder infections. These data would agree with the data observed in the SARS‐CoV2 positive IS cohort, as most patients exhibit a mild SARS‐CoV2 infection (Asakura and Ogawa 2021).

In addition, we have measured the concentration of various fibrinolytic markers. tPA is a fibrinolytic protein that mediates plasmin formation through a lysine binding site, which in turn promotes fibrin removal and degradation. Recombinant tPA (rtPA) is currently the only thrombolytic agent able to induce the dissolution of the thrombus post‐IS. A total of seven patients from the SARS‐CoV2 negative IS and two SARS‐CoV2 positive IS patients were treated with rtPA. However, rtPA has a half‐life of less than 10 min and is not expected to be detectable at the time of the blood sampling (Murray et al. 2010). The SARS‐CoV2 positive IS cohort presented increased plasma levels of endogenous tPA compared to non‐stroke controls, which may indicate a degree of fibrinolysis, while the SARS‐CoV2 negative IS cohort did not show any changes in tPA. Furthermore, PAI‐1, a serine protein inhibitor, which is a physiological inhibitor of tPA, was found unchanged in all experimental cohorts. These data suggest that there is no inhibition of fibrinolysis after IS independently from the infection. A robust fibrinolysis inhibition has been reported in experimental models of IS (Denorme et al. 2016) and a cohort of SARS‐CoV2 patients (Baycan et al. 2023), which is not reflected in our study. The discrepancy may be due to the inability to discriminate between either tPA and PAI‐1 antigens or tPA/PAI‐1 complexes using LEGENDPlex assays.

Finally, plasma levels of MMP‐2 and ICAM‐1 were found to be elevated in the SARS‐CoV2 negative IS patients. MMP‐2 is an extracellular matrix (ECM) protein involved in the degradation of several ECM proteins including collagen IV, fibronectin, and gelatin. Human serum studies have previously highlighted that MMP‐2 is unchanged in the acute phase post‐stroke. Interestingly, MMP‐2 is reported to increase from a week to 4 months post‐stroke and may play a protective and remodeling role (Lucivero et al. 2007; Clark et al. 1997). In addition, another study on a small cohort of patients infected with SARS‐CoV2 has shown lowered levels of MMP‐2, which may explain the absence of change in the SARS‐CoV‐2‐positive IS patients. The decrease after the infection may counterbalance the increase that would be observed after IS, leading to an overall absence of change (Avila‐Mesquita et al. 2021). ICAM‐1 is an important protein involved in the recruitment of leukocytes to the endothelium and plays a pivotal role in the inflammatory progression. sICAM‐1 levels have been shown to be elevated post‐stroke in clinical and preclinical studies, with sICAM‐1 being associated with the severity and negative outcome of IS, in line with the findings of our study (Moller et al. 2015). Interestingly, in SARS‐CoV2, sICAM‐1 was found to be low in the initial 2 weeks post‐infection and then increased in the chronic phase of the infection. Similar to MMP‐2, the plasma levels of sICAM‐1 may be inherently low in the acute phase of the infection which may counterbalance the increase observed in the IS (Smith‐Norowitz et al. 2021).

The current study has several limitations, largely as a result of the extremely challenging situation for clinical research during the COVID‐19 pandemic. Most notably, the study has a relatively small number of patients in the different experimental cohorts (*n* = 22–24), meaning subtle thromboinflammatory changes may be harder to detect. Also, patients in this study had relatively mild strokes and SARS‐CoV2 infections, which likely affects the magnitude of any thromboinflammatory changes. In addition, we only quantified admission samples, meaning we were unable to assess thromboinflammatory changes over a longer period of time and how any changes might associate with the outcome. This is a retrospective study; the SARS‐CoV2 positive IS cohort being recruited before the SARS‐CoV2 negative IS cohort. All patients in the SARS‐CoV2 negative IS cohort were free from RTIs in the two weeks preceding their admission in the stroke unit. However, a patient in the SARS‐CoV2 negative IS cohort was commenced on a course of antibiotics 2 days after admission in the stroke unit, indicating a post‐stroke infection. Finally, the study lacks a SARS‐CoV2 only cohort, which would have allowed us to underpin the extent and magnitude of the thromboinflammatory response caused by infection alone. However, there have been extensive reports published on thromboinflammatory changes in SARS‐CoV2 infection patients, and where relevant, these are referred to throughout this manuscript, changes being compared to those that we observed in our respective cohorts. Overall, despite these limitations, we believe this to be the first study to provide data on thromboinflammatory changes in IS patients with the proven presence or absence of SARS‐CoV2 infection, with very well‐matched experimental cohorts based on age, NIHSS, and sex.

Overall, our study has highlighted that the thromboinflammatory response post‐stroke is affected by the preceding SARS‐CoV2 infection by enhancing the VWF/ADAMTS13 axis imbalance and does not elevate to the same magnitude the protein levels of MMP‐2, sICAM‐1, and FIX. In conclusion, this study provides more insights on how infection affects molecular thromboinflammatory changes post‐stroke.

## Author Contributions

**Nadim Luka**: conceptualization, data curation, formal analysis, visualization, writing–original draft, methodology, investigation. **Kieron South**: conceptualization, writing–original draft, methodology, investigation, supervision, writing–review and editing, funding acquisition. **Graham Coutts**: project administration, methodology. **Ioana–Emilia Mosneag**: supervision, writing–review and editing. **Rachel Jones**: investigation, data curation, writing–review and editing. **Amanda J. Unsworth**: investigation, data curation, writing‐review and editing. **Mehwish Younas**: project administration, data curation. **Amy Bradley**: project administration, writing–review and editing, data curation. **Siew Yan Wong**: project administration, data curation. **Ellen Collins**: project administration, data curation. **Chloe Quigley**: project administration, data curation. **Sean B. Knight**: writing–review and editing, project administration, data curation. **Barry W. McColl**: project administration, writing–review and editing, data curation. **Laura McCulloch**: project administration, writing–review and editing, data curation. **John Grainger**: project administration, data curation. **Craig J. Smith**: conceptualization, data curation, methodology, funding acquisition, supervision, project administration, writing–review and editing. **Stuart M. Allan**: conceptualization, data curation, methodology, supervision, project administration, writing–review and editing, funding acquisition.

## Ethics Statement

Ethical approval was obtained prior to the start of the study.

## Conflicts of Interest

The authors declare no conflict of interest.

### Peer Review

The peer review history for this article is available at https://publons.com/publon/10.1002/brb3.70348.

## Data Availability

Data are available upon request.

## References

[brb370348-bib-0001] Abdul, S. , J. Boender , J. Malfliet , et al. 2017. “Plasma Levels of Plasminogen Activator Inhibitor‐1 and Bleeding Phenotype in Patients With Von Willebrand Disease.” Haemophilia 23, no. 3: 437–443. 28306198 10.1111/hae.13206

[brb370348-bib-0002] Andersson, H. M. , B. Siegerink , B. M. Luken , et al. 2012. “High VWF, Low ADAMTS13, and Oral Contraceptives Increase the Risk of Ischemic Stroke and Myocardial Infarction in Young Women.” Blood 119, no. 6: 1555–1560. 22110247 10.1182/blood-2011-09-380618

[brb370348-bib-0003] Arce, N. A. , W. Cao , A. K. Brown , et al. 2021. “Activation of Von Willebrand Factor via Mechanical Unfolding of Its Discontinuous Autoinhibitory Module.” Nature Communications 12, no. 1: 2360. 10.1038/s41467-021-22634-x PMC8060278 33883551

[brb370348-bib-0004] Asakura, H. , and H. Ogawa . 2021. “COVID‐19‐Associated Coagulopathy and Disseminated Intravascular Coagulation.” International Journal of Hematology 113, no. 1: 45–57. 33161508 10.1007/s12185-020-03029-y PMC7648664

[brb370348-bib-0005] Avila‐Mesquita, C. D. , A. E. S. Couto , L. C. B. Campos , et al. 2021. “MMP‐2 and MMP‐9 Levels in Plasma Are Altered and Associated With Mortality in COVID‐19 Patients.” Biomedicine & Pharmacotherapy 142: 112067. 34449310 10.1016/j.biopha.2021.112067 PMC8376652

[brb370348-bib-0006] Baycan, O. F. , H. A. Barman , F. Bolen , et al. 2023. “Plasminogen Activator Inhibitor‐1 Levels as an Indicator of Severity and Mortality for COVID‐19.” Northern Clinics of İstanbul 10, no. 1: 1–9. 36910430 10.14744/nci.2022.09076 PMC9996651

[brb370348-bib-0007] Bernardo, A. , C. Ball , L. Nolasco , J. F. Moake , and J. F. Dong . 2004. “Effects of Inflammatory Cytokines on the Release and Cleavage of the Endothelial Cell‐Derived Ultralarge Von Willebrand Factor Multimers Under Flow.” Blood 104, no. 1: 100–106. 15026315 10.1182/blood-2004-01-0107

[brb370348-bib-0008] Chi, L. , Y. Li , L. Stehno‐Bittel , et al. 2001. “Interleukin‐6 Production by Endothelial Cells via Stimulation of Protease‐Activated Receptors Is Amplified by Endotoxin and Tumor Necrosis Factor‐Alpha.” Journal of Interferon & Cytokine Research 21, no. 4: 231–240. 11359654 10.1089/107999001750169871

[brb370348-bib-0009] Chion, C. K. , C. J. Doggen , J. T. Crawley , D. A. Lane , and F. R. Rosendaal . 2007. “ADAMTS13 and Von Willebrand Factor and the Risk of Myocardial Infarction in Men.” Blood 109, no. 5: 1998–2000. 17053057 10.1182/blood-2006-07-038166

[brb370348-bib-0010] Clark, A. W. , C. A. Krekoski , S. S. Bou , K. R. Chapman , and D. R. Edwards . 1997. “Increased Gelatinase A (MMP‐2) and Gelatinase B (MMP‐9) Activities in Human Brain After Focal Ischemia.” Neuroscience Letters 238, no. 1‐2: 53–56. 9464653 10.1016/s0304-3940(97)00859-8

[brb370348-bib-0011] Denorme, F. , P. Kraft , I. Pareyn , et al. 2017. “Reduced ADAMTS13 Levels in Patients With Acute and Chronic Cerebrovascular Disease.” PLoS One 12, no. 6: e0179258. 28591212 10.1371/journal.pone.0179258 PMC5462472

[brb370348-bib-0012] Denorme, F. , K. Martinod , A. Vandenbulcke , et al. 2021. “The von Willebrand Factor A1 Domain Mediates Thromboinflammation, Aggravating Ischemic Stroke Outcome in Mice.” Haematologica 106, no. 3: 819–828. 32107335 10.3324/haematol.2019.241042 PMC7927893

[brb370348-bib-0013] Denorme, F. , K. Vanhoorelbeke , and S. F. De Meyer . 2019. “Von Willebrand Factor and Platelet Glycoprotein Ib: A Thromboinflammatory Axis in Stroke.” Frontiers in Immunology 10: 2884. 31921147 10.3389/fimmu.2019.02884 PMC6928043

[brb370348-bib-0014] Denorme, F. , T. Wyseure , M. Peeters , et al. 2016. “Inhibition of Thrombin‐Activatable Fibrinolysis Inhibitor and Plasminogen Activator Inhibitor‐1 Reduces Ischemic Brain Damage in Mice.” Stroke: A Journal of Cerebral Circulation 47, no. 9: 2419–2422. 10.1161/STROKEAHA.116.014091 27470988

[brb370348-bib-0015] Di Napoli, M. , and P. Singh . 2009. “Is Plasma Fibrinogen Useful in Evaluating Ischemic Stroke Patients?: Why, How, and When.” Stroke: A Journal of Cerebral Circulation 40, no. 5: 1549–1552. 10.1161/STROKEAHA.108.537084 19299637

[brb370348-bib-0016] Grassle, S. , V. Huck , K. I. Pappelbaum , et al. 2014. “Von Willebrand Factor Directly Interacts With DNA From Neutrophil Extracellular Traps.” Arteriosclerosis, Thrombosis, and Vascular Biology 34, no. 7: 1382–1389. 24790143 10.1161/ATVBAHA.113.303016

[brb370348-bib-0017] Hafez, W. , M. A. Ziade , A. Arya , et al. 2022. “Reduced ADAMTS13 Activity in Correlation With Pathophysiology, Severity, and Outcome of COVID‐19: A Retrospective Observational Study.” International Journal of Infectious Diseases 117: 334–344. 35167969 10.1016/j.ijid.2022.02.019 PMC8839807

[brb370348-bib-0018] Hou, H. Q. , X. L. Xiang , Y. S. Pan , et al. 2021. “Baseline or 90‐Day Fibrinogen Levels and Long‐Term Outcomes After Ischemic Stroke or TIA: Results From the China National Stroke Registry III.” Atherosclerosis 337: 35–41. 34757269 10.1016/j.atherosclerosis.2021.10.002

[brb370348-bib-0019] King, D. , R. Wittenberg , A. Patel , Z. Quayyum , V. Berdunov , and M. Knapp . 2020. “The Future Incidence, Prevalence and Costs of Stroke in the UK.” Age and Ageing 49, no. 2: 277–282. 31957781 10.1093/ageing/afz163 PMC7047821

[brb370348-bib-0020] Knight, R. , V. Walker , S. Ip , et al. 2022. “Association of COVID‐19 With Major Arterial and Venous Thrombotic Diseases: A Population‐Wide Cohort Study of 48 Million Adults in England and Wales.” Circulation 146, no. 12: 892–906. 36121907 10.1161/CIRCULATIONAHA.122.060785 PMC9484653

[brb370348-bib-0021] Ladikou, E. E. , H. Sivaloganathan , K. M. Milne , et al. 2020. “Von Willebrand Factor (vWF): Marker of Endothelial Damage and Thrombotic Risk in COVID‐19?” Clinical Medicine 20, no. 5: e178–e182. 32694169 10.7861/clinmed.2020-0346 PMC7539718

[brb370348-bib-0022] Lin, G. , M. Hu , J. Song , et al. 2021. “High Fibrinogen to Albumin Ratio: A Novel Marker for Risk of Stroke‐Associated Pneumonia?” Frontiers in Neurology 12: 747118. 35095715 10.3389/fneur.2021.747118 PMC8792987

[brb370348-bib-0023] Liu, Y. , H. Chen , K. Zhao , W. He , S. Lin , and J. He . 2019. “High Levels of Plasma Fibrinogen Are Related to Post‐Stroke Cognitive Impairment.” Brain and Behavior 9, no. 10: e01391. 31475471 10.1002/brb3.1391 PMC6790326

[brb370348-bib-0024] Lucivero, V. , M. Prontera , D. M. Mezzapesa , et al. 2007. “Different Roles of Matrix Metalloproteinases‐2 and ‐9 After Human Ischaemic Stroke.” Neurological Sciences 28, no. 4: 165–170. 17690845 10.1007/s10072-007-0814-0

[brb370348-bib-0025] Mancini, I. , L. Baronciani , A. Artoni , et al. 2021. “The ADAMTS13‐von Willebrand Factor Axis in COVID‐19 Patients.” Journal of Thrombosis and Haemostasis 19, no. 2: 513–521. 33230904 10.1111/jth.15191 PMC7753796

[brb370348-bib-0026] Mann, E. R. , M. Menon , S. B. Knight , et al. 2020. “Longitudinal Immune Profiling Reveals Key Myeloid Signatures Associated With COVID‐19.” Science Immunology 5, no. 51: eabd6197. 32943497 10.1126/sciimmunol.abd6197 PMC7857390

[brb370348-bib-0027] Marchetti, M. , P. Gomez‐Rosas , L. Russo , et al. 2022. “Fibrinolytic Proteins and Factor XIII as Predictors of Thrombotic and Hemorrhagic Complications in Hospitalized COVID‐19 Patients.” Frontiers in Cardiovascular Medicine 9: 896362. 35757331 10.3389/fcvm.2022.896362 PMC9226333

[brb370348-bib-0028] Masias, C. , and S. R. Cataland . 2018. “The Role of ADAMTS13 Testing in the Diagnosis and Management of Thrombotic Microangiopathies and Thrombosis.” Blood 132, no. 9: 903–910. 30006329 10.1182/blood-2018-02-791533

[brb370348-bib-0029] Moller, K. , C. Posel , A. Kranz , et al. 2015. “Arterial Hypertension Aggravates Innate Immune Responses After Experimental Stroke.” Frontiers in Cellular Neuroscience 9: 461. 26640428 10.3389/fncel.2015.00461 PMC4661280

[brb370348-bib-0030] Muia, J. , J. Zhu , G. Gupta , et al. 2014. “Allosteric Activation of ADAMTS13 by Von Willebrand Factor.” Proceedings of the National Academy of Sciences of the United States of America 111, no. 52: 18584–18589. 25512528 10.1073/pnas.1413282112 PMC4284596

[brb370348-bib-0031] Murray, V. , B. Norrving , P. A. Sandercock , A. Terent , J. M. Wardlaw , and P. Wester . 2010. “The Molecular Basis of Thrombolysis and Its Clinical Application in Stroke.” Journal of Internal Medicine 267, no. 2: 191–208. 20175866 10.1111/j.1365-2796.2009.02205.x

[brb370348-bib-0032] Newnham, M. , K. South , M. Bleda , et al. 2019. “The ADAMTS13‐VWF Axis Is Dysregulated in Chronic Thromboembolic Pulmonary Hypertension.” European Respiratory Journal 53, no. 3: 1801805. 30655285 10.1183/13993003.01805-2018 PMC6437602

[brb370348-bib-0033] Oh, S. E. , and N. S. Parikh . 2022. “Recent Advances in the Impact of Infection and Inflammation on Stroke Risk and Outcomes.” Current Neurology and Neuroscience Reports 22, no. 3: 161–170. 35235168 10.1007/s11910-022-01179-6 PMC8889053

[brb370348-bib-0034] Paganini‐Hill, A. , E. Lozano , G. Fischberg , et al. 2003. “Infection and Risk of Ischemic Stroke: Differences Among Stroke Subtypes.” Stroke: A Journal of Cerebral Circulation 34, no. 2: 452–457. 10.1161/01.str.0000053451.28410.98 12574559

[brb370348-bib-0035] Perry, R. J. , C. J. Smith , C. Roffe , et al. 2021. “Characteristics and Outcomes of COVID‐19 Associated Stroke: A UK Multicentre Case‐Control Study.” Journal of Neurology, Neurosurgery, and Psychiatry 92, no. 3: 242–248. 33154179 10.1136/jnnp-2020-324927

[brb370348-bib-0036] Petri, B. , A. Broermann , H. Li , et al. 2010. “Von Willebrand Factor Promotes Leukocyte Extravasation.” Blood 116, no. 22: 4712–4719. 20716766 10.1182/blood-2010-03-276311

[brb370348-bib-0037] Peyvandi, F. , I. Garagiola , and L. Baronciani . 2011. “Role of Von Willebrand Factor in the Haemostasis.” Blood Transfusion 9, no. S2: s3–s8. 21839029 10.2450/2011.002S PMC3159913

[brb370348-bib-0038] Schick, P. K. , J. Walker , B. Profeta , L. Denisova , and V. Bennett . 1997. “Synthesis and Secretion of Von Willebrand Factor and Fibronectin in Megakaryocytes at Different Phases of Maturation.” Arteriosclerosis, Thrombosis, and Vascular Biology 17, no. 4: 797–801. 9108796 10.1161/01.atv.17.4.797

[brb370348-bib-0039] Smith‐Norowitz, T. A. , J. Loeffler , Y. M. Norowitz , and S. Kohlhoff . 2021. “Intracellular Adhesion Molecule‐1 (ICAM‐1) Levels in Convalescent COVID‐19 Serum: A Case Report.” Annals of Clinical and Laboratory Science 51, no. 5: 730–734. 34686518

[brb370348-bib-0040] South, K. , O. Saleh , E. Lemarchand , et al. 2022. “Robust Thrombolytic and Anti‐Inflammatory Action of a Constitutively Active ADAMTS13 Variant in Murine Stroke Models.” Blood 139, no. 10: 1575–1587. 34780600 10.1182/blood.2021012787 PMC11017955

[brb370348-bib-0041] Sui, J. , D. F. Noubouossie , S. Gandotra , and L. Cao . 2021. “Elevated Plasma Fibrinogen Is Associated With Excessive Inflammation and Disease Severity in COVID‐19 Patients.” Frontiers in Cellular and Infection Microbiology 11: 734005. 34414135 10.3389/fcimb.2021.734005 PMC8369350

[brb370348-bib-0042] Takacs, T. T. , A. J. Berki , P. P. Bojti , et al. 2023. “The Impact of SARS‐CoV‐2 Infection on the Outcome of Acute Ischemic Stroke‐A Retrospective Cohort Study.” PLoS One 18, no. 3: e0282045. 36862706 10.1371/journal.pone.0282045 PMC9980769

[brb370348-bib-0043] Taylor, A. , C. Vendramin , D. Singh , M. M. Brown , and M. Scully . 2020. “Von Willebrand Factor/ADAMTS13 Ratio at Presentation of Acute Ischemic Brain Injury Is Predictive of Outcome.” Blood Advances 4, no. 2: 398–407. 31990334 10.1182/bloodadvances.2019000979 PMC6988400

[brb370348-bib-0044] Thangaraju, K. , U. Katneni , I. J. Akpan , et al. 2021. “The Impact of Age and BMI on the VWF/ADAMTS13 Axis and Simultaneous Thrombin and Plasmin Generation in Hospitalized COVID‐19 Patients.” Frontiers in Medicine 8: 817305. 35087853 10.3389/fmed.2021.817305 PMC8786628

[brb370348-bib-0045] Vagionas, D. , D. D. Papadakis , M. Politou , A. Koutsoukou , and I. Vasileiadis . 2022. “Thromboinflammation in Sepsis and Heparin: A Review of Literature and Pathophysiology.” In Vivo 36, no. 6: 2542–2557. 36309378 10.21873/invivo.12991 PMC9677778

[brb370348-bib-0046] Yang, X. Y. , S. Gao , J. Ding , Y. Chen , X. S. Zhou , and J. E. Wang . 2014. “Plasma D‐Dimer Predicts Short‐Term Poor Outcome After Acute Ischemic Stroke.” PLoS One 9, no. 2: e89756. 24587013 10.1371/journal.pone.0089756 PMC3933671

[brb370348-bib-0047] Zhang, L. , C. Zhang , Y. Luo , and N. Tang . 2021. “Predictive Value of Coagulation Factor XIII on Bleeding Risk in Ischemic Stroke Patients Treated With Intravenous Thrombolysis.” Annals of Palliative Medicine 10, no. 7: 7579–7586. 34353045 10.21037/apm-21-1174

